# Cultural persistence and the ‘herbal medicine paradox’: Evidence from European data

**DOI:** 10.1177/13591053241237031

**Published:** 2024-04-02

**Authors:** Joan Costa-Font, Azusa Sato

**Affiliations:** 1 London School of Economics and Political Science, LSE, UK; 2 Asian Development Bank, Manila

**Keywords:** cultural persistence, culture, first and second-generation migrants, herbal medicines, intergenerational transmission, secondary analysis of migration data

## Abstract

The use of herbal or traditional medicines has survived the proliferation of modern medicine. The phenomenon has been labeled as the ‘herbal medicines paradox’ (HMP). We study whether such HMP hypothesis can be explained by the persistence of attitudes across cultural boundaries. We undertake a secondary analysis of individual-level migration data to test the persistence of the use of herbal medicines in relation to norms in the person’s country of birth (or home country). We study the association between attitudes towards herbal medicine treatments of both first (*N* = 3630) and second-generation (*N* = 1618) immigrants in 30 European countries, and the average attitudes of their sending country origins. We find robust evidence of an association that is stronger for the second-generation migrants. We document a stronger effect among maternal than paternal lineages, as well as significant heterogeneity based on migrants’ country of origin. Our estimates are robust to different sample analysis. Our estimates are consistent with a cultural explanation for the HMP.

## Introduction

The rapid progress of modern medicine has not fully supplanted traditional, herbal, or alternative treatments. Herbal treatments have continued to be used, even though health systems in Europe exclude such herbal treatments from their coverage (and patients therefore often pay out of pocket). This phenomenon has been labeled as the ‘herbal medicines paradox’ (HMP) (Costa-Font and Sato, 2017), and its behavioural mechanisms are still not well understood. The European Commission has adopted a Directive, the ‘Traditional Herbal Medicinal Products Directive’ (THMPD), to begin properly regulating the sector as a result of the scale of the alternative medicine sectors. Herbal or alternative medicines are an important part of health care treatment, and according to Rivera et al. (2013), an estimated 80% of the world’s population takes herbal medicines to treat a range of medical ailments. Access to traditional medicines is common in several countries that differ in cultural and religious ascription such as Chinese medicine, Unani medicine, European Traditional Medicine, and Brazilian medicine among others.

Decision-making explanations for the HMP include (i) the role of patient dissatisfaction with modern treatments, (ii) the fact that modern medicine is perceived as expert-driven, and provides limited patient control, as well as (iii) the role of individual values and beliefs, and worldviews (Astin, 1998; Sato and Costa-Font, 2014). Although such herbal treatments are far from perfect substitutes for modern treatments, their use continues to thrive over time. One potential explanation put forward by Salmon (2022) is that modern medicine does not adequately address the needs of individuals with a wide range of chronic conditions. However, estimates suggest that the proliferation of chronic conditions does not explain attitudes to medicine uptake (Astin, 1998). Today, it is unclear whether personalised treatments of modern medicine provide any less individual control than modern personalised treatment. In fact, in some Asian countries, modern medical professionals incorporate traditional medicines in their treatments and the general populace use them regularly. For instance, Hon et al. (2005) document that a majority of medical students in Hong Kong use traditional medicines without medical recommendation or knowledge of potential side effects.

Of the three classical explanations for the use of traditional medicines, the role of beliefs and values stands as the most plausible one. Nonetheless, health care beliefs are not typically individually crafted; instead, they are commonly moulded by cultural influences and practices that have evolved within communities with shared pasts and encounters (Picking and Vandebroek, 2019). These cultural influences are deeply ingrained in identities and upheld by societal standards (Uhlaner, 1989: 254). This study aims to delve into the impact of portable cultural dimensions on shaping individual perspectives on traditional medicine healthcare.^1^

Given that health is a relational good, culture plays a primordial role in influencing preferences for health care treatments. However, so far, the influence of culture in such preferences is proxied by the role of some attitudes and beliefs. Indeed, some research documents that ‘entrenched cultural beliefs’ correlate with attitudes to traditional medicines in the US (Astin, 1998) and in the Philippines and Ghana (Costa-Font and Sato, 2017). However, such research does not isolate the effect of culture from the context, or institutions in which such beliefs are formed. A common way to disentangle the effect of cultural values from other contextual effects lies in examining the attitudes of both first- and second-generation migrants. Studying migrant attitudes allows us to study the portable dimensions of culture that individuals take with them when they migrate. This allows empirically disentangling the effect of culture from the institutions and country contexts they are subject to. This is especially the case of second-generation migrants. Migrants carry their values with them, which are then transmitted inter-generationally to the second generation raised within the host institutions. Consequently, when there is sufficient diversity in both migrants’ homes and host countries, it becomes feasible to analyse the correlation between attitudes in both types of countries.

This paper undertakes a secondary analysis of migration data used to test the persisting use of herbal medicines in relation to norms in the person’s country of birth or home country. Such evidence can help to understand the extent to which attitudes to herbal medicine have *deep cultural roots*, namely cultural reference points that can be traced back to specific solutions to past medical problems that remain inertial over time, irrespective of technological advances. More generally, attitudes are formed from processes of socialisation around some collective traits which are often picked up during childhood and tend to remain stable over time (DiMaggio, 1997). Hence, we hypothesise that the latter could explain attitudes towards traditional or herbal medicines and the use of care provided by healers (Kleinman et al., 1978). Understanding and examining how culturally persistent are attitudes to traditional medicines, is an important research question that is far from settled. This is the goal of this paper.

So far, we know that beliefs, values, and social norms of each cultural setting influence individuals’ behaviour (Hofstede, 2001), which extends to health care related behaviours such as organ donation (Gillum and Masters, 2010; Graf et al., 2024), and as we argue here, the use of traditional medicines. This is important given that culture influences both the perceptions of, and trust in, the health system. Cultural reference points can explain the historical interactions that have left their mark on today’s health care decisions, even when such decisions are neither optimal nor suitable to today’s needs.

Cultural reference points can explain differences in utilization that cannot be attributed to illness prevalence or medical need, and underpin the evidence of large variability in clinical practices, as well as ethnic or religious differences in health care trust. Examples include Wennberg (2002) who documents evidence of a large variability in health outcomes and practices across the states in the United States even though economic incentives are similar. Similarly, russian immigrants frequently view U.S. medical care with a degree of mistrust (McLaughlin and Braun, 1998). Other examples of cultural influences include observant Muslims’ resistance to eating or taking medications during the daytime hours of Ramadan, or Hinduist acceptance of pain and suffering because of karma.

Given that culturally formed values and attitudes are geographically portable traits across time by migrants, it is possible to examine how they vary across generations of migrants in the host country. For instance, some evidence documents that African Americans view receiving health care as a humiliating experience (Spector, 2000), which is the result of treatment within the context of Western medicine in the US (e.g. the infamous Tuskegee Study) that has led to significant mistrust in the medical community (Jaiswal and Halkitis, 2019).

Although culturally formed social norms play a significant role in the delivery of healthcare and can influence health system efficiency, we still know little about the cultural persistence of such behaviors. Hence, understanding cultural persistence of attitudes and behaviours is crucial to predicting the patterns of health care use (Salant and Lauderdale, 2003), and how best to intervene and change individual actions.

In this paper, we draw on a secondary analysis of survey data to examine whether first-generation migrants’ attitudes towards the use of herbal medicines are associated with the preferences of their country of origin. We measure cultural persistence as the extent to which migrants’ health-related preferences are more akin to those of their country of origin or ascendance (Salant and Lauderdale, 2003). Examining data from migrants allows us to isolate the effect of culture from institutions, and hence address omitted variable bias from unobservable variables such as measures of health knowledge (which often affect the inappropriate use of medicine treatments too), parental health, and parental-specific characteristics. To do so, we follow the so-called ‘epidemiological approach’, which relates behaviors from second-generation immigrants to that of the average (social norms) of their birth countries (Fernandez, 2011; Luttmer and Singhal, 2011). Immigrants present a quasi-natural experiment to study evolving cultural norms, and our dataset contains a rich set of controls for confounding variables including country-fixed effects (country income) as well as potential country and time-specific shocks (which may differ across countries). Accordingly, our first hypothesis is the following:

**H1:** Attitudes towards the use of herbal treatments are culturally persistent, namely associated with cultural reference points that migrant individuals hold from their sending country.

Given that individuals are subject to acculturation processes, we expect a time-varying association between attitudes towards herbal medicines in sending and host countries over time, and specifically, we expect to find differences between maternal and paternal migration lineages consistently with previous research (Silva Júnior et al., 2014). Furthermore, we specifically focus on second-generation migrants who have been raised in the same institutions as natives.^2^ Different country cultures reflect different social norms and expectations (McLaughlin and Braun, 1998. We build on Alba and Nee (1997) to explore heterogeneity in cultural contexts and beliefs over time across first and second-generation immigrants and different countries. However, a key question refers to how culturally persistent preferences for herbal medicines are.^3^ We control for a variety of socioeconomic and demographic factors, as well as time and country fixed effects, and we report standardised coefficients, to compare means across the first and second generations. Hence, our second hypothesis is formulated as follows:

**H2:** Cultural persistence in attitudes to herbal medicines differs both between first- and second-generation migrants and by maternal and paternal lineage, and by time.

To test this hypothesis, we draw on a secondary data analysis to identify the association between migrant individuals (both first and second generation) with that of their sending country. It is important to note that there is no reverse causality as the child’s health care is unlikely to affect health care use in the father or mother’s country of origin.

We further test the hypothesis that time spent residing in the host country increases social contact and familiarity with the healthcare system for first-generation migrants, and we formulate the following final hypothesis:

**H3:** Time in the country (acculturation), migrant selection, and European citizenship of individuals from sending countries influence the persistence in the cultural transmission of herbal medicine attitudes.

We examine whether the time spent residing in the host country increases social contact and familiarity with the healthcare system for first-generation migrants. However, empirically a problem that needs to be addressed is that of migrant selection and more specifically the so-called ‘healthy migrant effect’ (Razum et al., 2000). That is, given that samples of migrants can differ from the rest of the population in key socio-economic and health dimensions one needs to account for the fact that examining evidence of such samples might provide different inference to that of the general population. Immigrant health is often found to be better than natives at the point of immigration (Frisbie et al., 2001; McDonald and Kennedy, 2004). However, in the European context, this does not apply, the only determinants of immigrant health refer to aspects that correlate with religion, whereby muslims have worse health than non-Muslims. Cultural persistence is largely dependent on patterns of socialisation and the capacity to network to reduce stress (Umberson and Montez, 2010). To control for potential selection problems, we carry out two additional tests. First, we condition on important health-related characteristics of migrants, which we refer to as ‘wellbeing controls’ in Table 1. Second, we examine the same specification for a subsample of European Union migrants who have the same rights in the period than in the country of origin, and hence, its composition is more likely to resemble that of natives. Second, citizens’ rights similarity is an important variable to control for, as the difference in rights could explain heterogeneous access to health care, which could both explain attitudes toward traditional medicines and could explain in turn the migrants’ selection.

**Table 1.** Association between attitudes to herbal medicines (HM) of first-generation migrants and those of the country of origin (CO) – baseline and with controls and interactions (OLS).

	I	II	III
HM Attitude (in CO)	0.864** (0.05)	0.713** (0.117)	0.671** (0.118)
Welfare	No	Yes	Yes
Socioeconomic	No	Yes	Yes
Country	No	No	Yes
Time in the country	No	No	Yes
Constant	0.335** (0.124)	1.026 (0.565)	0.517 (0.60)
Observations	3630	1531	1531
*R*-squared	0.109	0.255	0.256

Note: The first column is the baseline specification (without controls; the second column includes all controls except interactions; the third column reflects time in the country. Controls include those proxying for welfare (whether hampered in daily activities by illness, disability, infirmary or mental problem); opinion on the state of health services in the country nowadays); whether feel discriminated; socioeconomic and demographic status (such as religious denomination); how long have lived in the country; whether belong to minority ethnic group in the country; the number of people in the household; gender; marital status; age; the number of years of education; main occupational activity; household net income quintile; opinion on the state of health services in their country of origin; feeling about household’s income nowadays, whether a citizen of the country, country variable; country income quintile (country quintile). Robust standard errors under coefficients, **significant at 1%.

## Framework

### Culture and health care use

Most studies looking at beliefs about herbal medicines explain how people’s interpretations of illness – and therefore reactions to disease – are driven by ‘culture’ (Borrell-Porta et al., 2017; Helman, 2007; Winkelman, 2008). Culture refers to ‘customary beliefs and values’ that ethnic, religious, and social groups transmit fairly unchanged from generation to generation’, and shape individual worldviews. Dein (2004) find that ethnicity (as proxying for minority groups) is significantly associated with the belief that ‘some (traditional) home remedies are still better than prescribed drugs for curing illnesses’. Similarly, Winkler et al. (2010) document that stronger beliefs (such as symptoms and causes) about traditional healing methods for epilepsy in Tanzania are held by men and influenced by tribe, religion, and non-urban location. Within modern medicines, there is also evidence suggesting that the cultural background is also significantly associated with health beliefs (Horne et al., 2004) and in turn, treatment-seeking patterns (Karasz and McKinley, 2007). However, examining patterns of cultural persistence in health care behaviors requires the consideration of different mechanisms, as below.

### Cultural norms

The idea that cultural beliefs linger and evolve is well documented but remains debated, nonetheless. Owusu-Daaku and Smith (2005) show that Ghanaian women who have moved to the UK uphold Ghanaian perspectives about health and illness while adapting to the British health system. Similarly, Adherence to original identity is an example of ‘adhesive adaptation’ or ‘acculturation’ which explains that new cultures and social relations integrate into existing cultural beliefs, and do not replace old beliefs (Hurh and Kim, 1984, 1990). This approach is consistent with more complex models in the psychology literature (Berry et al., 1989) which focus on looking at the extent to which individuals separate from modern health care practices and consider alternative types of acculturations such as full assimilation, integration, separation and alienation responses to the immigration experience.

### The speed of cultural persistence

Time in a country can lead individuals to shape migrant preferences to be more in consonance with those of the host country. Redstone Akresh (2009) finds a positive relationship between time spent in the country and physician and dental visits even after controlling for several predisposing characteristics, self-assessed and physician-diagnosed needs. As the number of years in a country rises, immigrants may become more comfortable with the local language and gain familiarity with the health care system, resulting in increased usage. Redstone Akresh (2009) also suggests that immigrants may seek health care less frequently if they hold jobs in places where health insurance is available, making health service utilisation a luxury rather than a necessity. The effects seem to be different as we examine second-generation migrants. Dinesen and Hooghe (2010) find that second-generation immigrants to Europe are more assimilated than the first-generation. However, it is unclear whether this applies across the board. Hence, this paper contributes to this literature by making use of evidence from the demand for herbal medicines.

## Materials and methods

### Data

We draw upon secondary data from the European Social Survey (ESS) a cross-section European dataset that contains waves for every 2 years from 2002 and refers to a repeated cross-section that overall provides a pooled sample of 50,000 respondents (available in this link: https://www.europeansocialsurvey.org), out of which we could identify a sample 3730 first- generation migrants and 1799 second-generation migrants. The data includes 30 participating countries, and the survey contains information about the respondent’s country of birth and that of his/her father and mother. Furthermore, the data contains records of first- and second-generation migrants from over 90 countries, and accordingly, individual-level data can be matched with health measures constructed at the country level from the World Values Survey. Country refers to a European member state, and citizenship refers to the country of residence, alongside European Union (EU) citizenship when the country belongs to the EU.

Another advantage of this data is that we can control for the country of origin and residence country income (GDP per capita), mainly obtained from the World Bank database. This strategy has been previously used by Lutmer and Singhal (2011) to study preference for redistribution.

### Measurement of attitudes to herbal medicines

Our dependent variable refers to a measure of attitudes towards herbal medicines defined as the response to a question on whether the respondent uses herbal remedies if a health problem arises (‘How often do you use herbal remedies if health problem’, (and potential responses include ‘Never or almost never’, ‘Some of the time’, ‘about half the time’, ‘most of the time’, ‘always or almost always’ and ‘don’t have health problem’).

The same dataset also contains information on the migration status of the respondents and their parents, which allows us to identify the country of origin of respondents and their parents. Individual-level data were then merged with health measures constructed at the country level and income data (GDP per capita), taken mainly from the World Bank database.^4^ Our dependent variable refers to attitudes towards herbal medicines defined as the response to a question on whether the respondent uses herbal remedies if a health problem, (and how often).

### Controls included

We use mean values for the home country (to capture cultural effects) for the following variables, namely attitudes to traditional medicine of both the respondent’s country of birth and well as the paternal country of birth. The baseline specification includes population weights and wave controls but no other controls. Then we have included controls that we classify as those proxying for welfare (whether hampered in daily activities by illness, or disability); whether feel discriminated against; socioeconomic and demographic status (gender, age, and household size) as well as religious denomination. Our data contains records on how long individuals have lived in-country and whether they belong to a minority ethnic group in-country; alongside educational attainment, we include main occupational activity and household net income quintile. Finally, to control for institutions, we include the opinion on the state of health services in their country of origin and their feeling about household income nowadays as well as citizenship information. Further details of all variables are available in Appendix 1. We restrict ourselves to migrants from the same countries covered in the ESS.

### Sample

From our master dataset, we have created two samples: one for the first generation (defined as people born in one country and moved to another) and another for the second generation (defined as children of first-generation immigrants – where parents are not born in the same country as the child). In the latter, we run different regressions depending on the specific lineage, one with the father lineage (country of origin) and one with the maternal lineage. The broad range of immigrants from various countries in the ESS reduces the concern that estimates are driven by the effect of the small number of home country backgrounds. We present the summary statistics in Table A1.^5^

**Figure 1.**
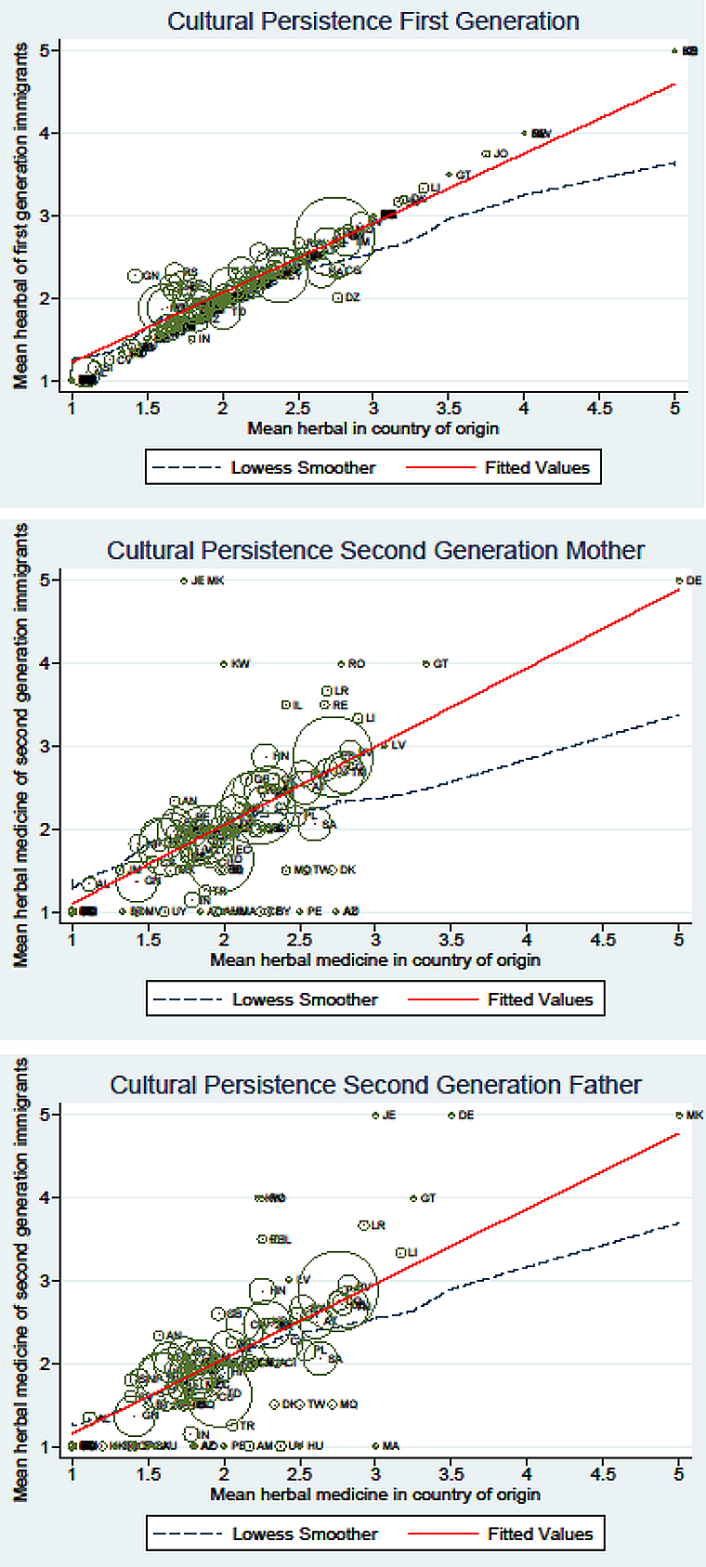
Correlation between attitudes of first and second-generation migrants and the mean attitudes of the sending country (country of origin). Note: This figure provides graphical evidence of the association between first and second-generation migrants’ preferences and that of their country of origin for first and second-generation migrants, and in the latter case, we differentiate between maternal and paternal lineage. The estimate and average size by country of origin are represented by the size of the circle.

Figure 1 plots the association of attitudes to herbal medicines with that of the home country (for first-generation migrants), and for second-generation migrants. Importantly, visual evidence is consistent with the hypothesis of robust cultural transmission, which appears to be stronger among second-generation migrants.

### Statistical analysis

Given that visual evidence might be sensible to the inclusion of several controls we examine the association between measures of use of herbal medicines of immigrants and that of their country of origin. We rely on the following specifications that measure cultural acculturation of first-generation migrants:

$$HM_{ij}=\;\rho {\overset{¯}{HM}}_{j}+\;\varphi X_{ij}+\gamma_{j}+\varepsilon_{ij}$$

Where $HM_{\mathrm{ij}}$ refers to herbal medicines attitudes of an individual from a home country j residing in the host country i, ${\bar{\mathrm{HM}}}_{j}$ refers to the home country j’s herbal medicine attitudes, and $X_{\mathrm{ij}}$ refers to individual specific controls that could upwardly bias the effect of cultural persistence, specifically can be divided in variables indicating proxy measures for the welfare and institutional controls and independent variables measuring i’s socioeconomic and demographic status. We include a parameter $\gamma_{\mathrm{jt}}$ that refers to a country-by-year fixed effect to account for the institutional setting and any other unobserved characteristics whether time-invariant or country-specific. Finally, $\varepsilon_{\mathrm{ij}}$ refers to the random term and may include country of origin fixed effects. All standard errors are clustered by the individual’s home country to avoid arbitrary serial correlations of error terms among individuals from the same home country. We have estimated linear probability models, but the results are replicated using both ordered probit and logit models. We have standardised the regression parameters to allow for comparing effects’ sizes and interpreting coefficients as ‘the effect of one standard deviation on herbal medicine use’.

Our coeffcient of interest refers to ρ, and measures the effect size reflected by different specifications. These association estimates reported in regression coefficients measure cultural persistence, after controlling for several other relevant factors, such as the time in the country. If ρ is close to zero, this would indicate no cultural preference. However, given that migrants have been raised under the institutions of the country of origin, ρ will pick up institutional effects and not the cultural effect alone. A common way to control local institutions, in addition to controls, includes focusing on second-generation migrants. In so doing, cultural transmission results from the parental transmission of preferences (from parents to children). We run two different specifications, one for the paternal lineage and one for the maternal lineage.

### The effect of migrant’s citizenship and selection

As a sensitivity analysis, we restrict our analysis of culture to migrants from a country other than where the survey was undertaken. This way we can precisely estimate the effect on the country of origin of migrants. Further, given that mobility restrictions within Europe are less stringent than between Europe and other parts of the world, and rights and regulations differ, we take a sample of migrants who are just from Europe to overcome potential sources of unobserved heterogeneity that could not be entirely controlled for with destination country fixed effects.

## Results

### First generation effects

Table 1 reports evidence of the cultural persistence of herbal medicine use. Results show strong association between the mean herbal medicines use in the country of origin and the migrant individual herbal medicine use suggesting very strong cultural persistence irrespective of the inclusion of controls. The standardized coefficient for the first generation suggests that a one standard deviation change in the country of origin use of herbal medicines is associated with a 0.331 percentage points increase in the attitudes towards own herbal medicine use. When we include controls (but no inclusion of time in the country), the effect size becomes 0.289, while it is 0.272 with interaction effects. That is, one standard deviation increase in average support for herbal medicines in the country of origin, increases 0.27 percentual points the intensity of the attitude towards herbal medicines by an average rating of 2.16 (12% increase).

### Second generation effects

In Table 2, we study second-generation effects by maternal and paternal lineage with and without controls. Results show very strong cultural effects across all lineage types. When we examine cultural persistence for the second generation irrespective of the lineage, the coefficient for the second generation is still significant and is of a smaller magnitude. That is, attitudes towards herbal medicines do not appear to soften from the first to the second generation, not for maternal lineage, nor both parent country lineages. Second-generation migrants’ attitudes are at last aligned with those of their grandparents, above and beyond first-generation effects. Formal tests of equality of coefficients suggest significance at a 5% level. When all controls including parental time in the country are included, we find that one standard deviation increase in average support for herbal medicines in the mother country or father country of origin increases support for herbal medicines by 0.285 percentage points (pp) and 0.239pp which corresponds to a 13% and 10% increase respectively.

**Table 2.** Association between attitudes to herbal medicines (HM) of second-generation migrants and those of the country of origin (CO) – maternal (M) and paternal (P) lineage, with and without controls (OLS).

	I	II	III	IV
HM Attitude (in CO) – P lineage	0.972** (0.133)	0.623** (0.169)		
HM Attitude (in CO) – M lineage			0.936** (0.112)	0.743** (0.263)
Welfare	No	Yes	No	Yes
Socioeconomic	No	Yes	No	Yes
Country	No	Yes	No	Yes
Parental time in country	No	Yes	No	Yes
Observations	1618	617	1637	623
*R*-squared	0.123	0.381	0.12	0.378

Note: The first column is the baseline specification (without controls; the second column includes all controls except reflecting parental time in the country; the third column includes such effects. Controls include those proxying for welfare (whether hampered in daily activities by illness, disability, infirmary or mental problem); opinion on the state of health services in the country nowadays); whether feel discriminated; socioeconomic and demographic status (such as religious denomination); how long have lived in country; whether belong to minority ethnic group in the country; the number of people in the household; gender; marital status; age; number of years of education; main occupational activity; household net income quintile; opinion on the state of health services in their country of origin; feeling about household’s income nowadays; whether a citizen of the country, country variable; country income quintile (country quintile). Robust standard errors, **significant at 1%.

### Citizenship and selection

In the first set of robustness checks, we look for gender differentials in Table 3. This is important because the literature has shown that assimilation effects can differ across men and women, however, the coefficient for women is significant and still suggests strong evidence of cultural persistence. Table 3 shows that whilst first-generation migrant men are more culturally linked to their country of origin than second-generation migrants, the opposite applies to women. Among second-generation migrants, the effect size is comparable to previous estimates, irrespectively of lineage and formal tests of equality of coefficient cannot reject the null hypothesis of equality.

**Table 3.** Robustness check (I): Association of Attitudes of towards Herbal Medicines (HM) of first- and second-generation migrants by gender–maternal (M) and paternal(P) lineage (OLS).

	I	II
	Male	Female
HM Attitude (in CO)	0.926** (0.164)	0.574** (0.121)
HM Attitude (in CO) – P lineage	0.452 (0.334)	0.858* (0.348)
HM Attitude (in CO) – M lineage	0.47* (0.24)	0.918** (0.34)

All controls are included as defined in Table 2. Robust standard errors are given under coefficients.

* Significant at 5%; **significant at 1%.

Given that citizenship status in European Union (EU) countries is not generally automatic after birth, institutions might still matter. Table 4 suggests that the effect changes when we examine the subsample of EU-born individuals who would not be constrained by citizenship constraints. We find evidence suggestive of cultural persistence but the effect among the second generation is weaker than that of the first. The latter is important because migrants might not have the same institutions in their country of residence across European countries. Restructuring the sample also allows us to overcome some potential unobserved heterogeneity that could not be entirely controlled with destination country fixed effects. In contrast, when we evaluate the effect only among residents in EU countries, we find evidence of a stronger cultural persistence among second-generation migrants. Formal testing of coefficient equality rejects the hypothesis of equality at a 1% level.

**Table 4.** Robustness check (II): Association of Attitudes of towards Herbal Medicines (HM) of first- and second-generation migrants by EU country or EU birth.

	I	II
	European Union born	European Union resident’s country
HM Attitude (in CO)	0.885** (0.144)	0.656** (0.110)
HM Attitude (in CO) – P lineage	0.428* (0.170)	0.617** (0.204)
HM Attitude (in CO) – M lineage	0.653* (0.246)	0.862** (0.236)

All controls are included as defined I Table. Robust standard errors are given under coefficients.

* Significant at 5%; **significant at 1%.

Finally, in the third set of robustness checks, regressions were rerun using a probit specification on a general preference question where dependent variables were binarianised accordingly. For the use of herbal medicines respondents were classed into those who answered, ‘never or almost never’ and the rest (‘some of the time’, ‘about half the time’, ‘most of the time’, ‘always or almost always’). Results are comparable to the previously shown ones.

## Discussion

We have studied the association between attitudes towards herbal treatments in the home and host country of first- and second-generation migrants using secondary analysis of migration data. The intensity of the association between attitudes provides relevant evidence of the persistence of cultural attitudes to herbal medicines. We document clear evidence of cultural persistence over time through to the second generation (**H1**). However, consistent with Silva Júnior et al. (2014) we show that cultural persistence appears to be stronger among the second generation respondents than the first, and among maternal lineages. That is, individuals’ attitudes toward first and second-generation migrants are significantly associated with that of their country of birth, or that of their parent’s country of birth, and stronger effects from the second generation than the first generation (**H2**). Furthermore, we find that time of residence in the host country *does not* make a difference and does not override the effect of the primary variables of interest, and that our estimates are not affected when we distinguish European citizenship and migrant selection (**H3**). However, we find that cultural persistence does differ by gender.

We have considered several robustness checks. First, we have examined differences in results owing to gender. Secondly, we have studied the role of selective migration by using subsamples of only EU countries. Thirdly, we have controlled for potential institutional heterogeneity in citizenship regulation by examining subsamples of those born in European Union countries, as citizens would have the same rights as the locally born, and only differ in their cultural values. Finally, we have rerun our analysis using probit rather than OLS, to consider the original nature of the data (which gives answers on a Likert scale) and probabilities of using or preferring one form of health care. All robustness checks confirm the previous results that suggest the presence of strong and persistent cultural effects underpinning herbal medicine use.

Our estimates suggest that where horizontal transmission (peer effects and country and influences in the country of residence) weighs more heavily in the formation of attitudes, one can expect some degree of convergence to the local population (assimilation). In contrast, when beliefs and cultural traits from vertical transmission (parental transmission) weigh more heavily in the formation of attitudes, then culture tends to be more persistent, and one would expect assimilation to slow down. Overall, our results suggest evidence of a strengthening of cultural persistence among the maternal lineage of second-generation migrants. However, estimates are sensitive to specific analyses on subsamples of migrants.

Our findings can be explained by the fact that attitudes towards health care are driven by some level of trust gap between health care practices in the sending and receiving countries, especially during the acculturation process to a new migrant country. Medication adherence tends to be lower for less threatening diseases, which are those that can be treated with herbal treatments (DiMatteo, 2004). Similarly, medication adherence is lower among individuals of lower socio-economic status and among minority racial backgrounds (Simoni et al., 2012), which again are more likely to use herbal treatments in many European countries. This points out the role of beliefs influenced by contextual factors that influence patient’s concerns about the medications, including the perception of those effects. Other explanations for limited trust in some settings refer to historical roots in prior medical abuse of minority populations (McQuaid and Landier, 2018)., and lower adherence to HIV medications (Dale et al., 2016).

Finally, our results highlight the importance of subjective factors, and cultural perceptions in the sending country, as opposed to objective measures of healthcare quality which typically are thought of as guiding health care decisions. Overall our estimates suggest that policy design on health and health care use needs to increasingly take into consideration (and filter) different culturally specific reactions and evaluations. Modern healthcare systems are progressively incorporating herbal treatments alongside, vitamins, and other complementary therapies to treat patients holistically and build trust. Consistently, one study from Jamaica shows 87% of herb users perceive herbal medicines to be more efficacious than modern medicines and therefore continue to utilise them (Clement et al., 2007). This evidence is consistent with a substantial body of research suggesting that though individuals are second-generation migrants, they interact mainly with similar peers, and they segregate strongly over settings. This means that these individuals do hold differences in health knowledge as they learn from and select similar peers through a process of co-evolution. Hence, cultural persistence might take place (or reinforce) through a social diffusion and learning process. Our results are consistent with this explanation.

### Limitations and study strengths

One of the potential concerns for future research to address is that of migrant’s selection on health status (McDonald and Kennedy, 2004; Palloni and Arias, 2004). However, tests of equality of means in our data suggest no evidence of health status differences across the sample of migrants and non-migrants. Once in the country, migrants are also likely to access better and more health care, which increases the probability of diagnosis and treatment (Jasso et al., 2004). That said, reliance on measures of attitudes inevitably leads to some level of imprecision, as attitude questions in surveys are generally vague, and do not allow an ideal and precise identification. This leaves open the question about the purpose of traditional medicine use, which we cannot respond fully to in this study. Finally, our sample considers 270 possible combinations of individuals from different host and receiving countries, but it is not a representative sample of all of them as this would require an extremely large sample which is unavailable to date. However, our interest is not to identify the cross-country effect but to purely exploit the variation in attitudes between sending and receiving countries that comes naturally in the sample and does not appear to be affected by obvious sample selection. That said, the diversity of the data can potentially be a limitation as it can entail small sample variation for each country, and hence potentially smaller power. However, in this study, we rely on a large sample of host and home countries. Finally, given that we use secondary data we cannot include the list of locations where herbal treatments are common, and we rely on a sample of European countries.

## Conclusion

This paper has undertaken a secondary analysis of survey data to test the cultural persistence of herbal medicines attitudes in relation to norms in the person’s country of birth or home country, which provide an explanation for the herbal medicine paradox (HMP). We have drawn upon a sample of European first and second-generation migrants, and we have accounted for an extensive set of controls. Importantly, evidence of the cultural persistence of attitudes towards herbal medicines suggests that the perceived efficacy of herbal medications is not necessarily aligned with that presented in biomedicine. The cultural persistence of herbal and alternative treatments seems to explain the HMP, namely that modern drugs have failed to completely displace herbal medicines. Consequently, health systems need to consider the right mix of modern and traditional medicines which are expected to coexist.

### Study implications

Although standard health decision frameworks assume that individuals make choices based on some form of the process of information updating rather than culturally determined health care practices, which takes time to build up, we show that reliance on traditional medicines and health care practices is more persistent than expected, far from adjusting in the first and second generation. Policy implications are suggestive that a strategy advocating ‘integration’ to place herbal medicines within a biomedical framework (World Health Organization, 2003) requires understanding the behavioural barriers that matter to people’s health care choices (e.g. trust) and considering them to design more user-friendly and culturally acceptable practices.

### Future directions

Future directions should identify whether the persistence of herbal and alternative treatments results from a rejection of modern medicines or merely the specific model of limited integration of herbal medicines in European health care systems. Furthermore, although we estimate effects up to the second-generation, it is unclear whether such cultural effects are observed beyond that. Access to data from third or further generations would allow a more precise analysis of the longer-term persistence of attitudes to herbal treatments. Finally, future research could estimate more precise measures of cultural norms and individual attitudes, perhaps using surveys and in-field experiments.

## Supplemental Material

sj-docx-1-hpq-10.1177_13591053241237031 – Supplemental material for Cultural persistence and the ‘herbal medicine paradox’: Evidence from European data Supplemental material, sj-docx-1-hpq-10.1177_13591053241237031 for Cultural persistence and the ‘herbal medicine paradox’: Evidence from European data by Joan Costa-Font and Azusa Sato in Journal of Health Psychology

## Notes

1. However, defining culture is inevitably complex, and that some dimensions of culture might not be fully captured.

2. It is important to state this does not mean that ‘social environments’ in which individuals are raised might still be different in settings where migrants are highly segregated, and migrants and natives move through different foci (schools/associations/churches/etc.).

3. Antecol (2000) finds that cultural effects persist until the second generation, while Borjas (1992) finds cultural persistence to be stronger for the second generation than any further generation of immigrants.

4. Available online at http://www.europeansocialsurvey.org/. Other sources of GDP per capita are available from IMF and World Bank sources (www.imf.org; www.worldbank.org).

5. As in other studies using the same data (Ljunge, 2014) we find that immigrants are similar to the general population on observable variables, with some differences in religion and education, which we control for along with some other controls.
