# Supplementary material for: Cultural persistence and the ‘herbal medicine paradox’: Evidence from European data
Source: J Health Psychol. 2024 Apr 2;30(2):171–85. doi: 10.1177/13591053241237031 (PMC11800700; doi:10.1177/13591053241237031)
Supplement: sj-docx-1-hpq-10.1177_13591053241237031 – Supplemental material for Cultural persistence and the ‘herbal medicine paradox’: Evidence from European data [file sj-docx-1-hpq-10.1177_13591053241237031.docx]

**Online Appendix**

**Table A1 Summary Variables (dependent variables and controls) in the main sample**

| **Variable name** | **Definition** | **No obs** | **Mean**  **(Standard dev)** |
| --- | --- | --- | --- |
| *Dependent variables* | | | |
| How often do you use herbal remedies if health problem | 1 Never or almost never  2 Some of the time  3 about half the time  4 most of the time  5 always or almost always  6 don’t have health problem | 46549 | 2.044  (1.194) |
| *Welfare controls* |  |  |  |
| whether hampered in daily activities by illness, disability, infirmary or mental problem | 1 Yes a lot  2 Yes to some extent  3 No | 48820 | 2.692  (0.580) |
| How happy are you? | 0 labelled ‘Extremely dissatisfied’, 10 labelled ‘Extremely satisfied’ | 48766 | 3.017  1.244 |
| opinion on state of health services in country nowadays | 0 (extremely bad) – 3 (extremely good) | 47672 | 1.391  (0.873) |
| Discrimination of respondent’s group: nationality | 0  1 | 49066 | 0.010 |
| *Social and demographic controls* | | | |
| religious denomination | 1 Roman Catholic  2 Protestant  3 Eastern Orthodox  4 Other Christian  5 Jewish and other non Christian  6 Islam  7 Eastern religion | 27448 |  |
| How long ago first came to live in country | 1 <20 years ago  2 20+ years ago  3 NA | 47483 | 2.878  (0.429) |
| Whether belong to minority ethnic group in country | 0 no  1 yes | 47956 | 0.042 |
| Number of people in household | 0 1-4  1 5+ | 49019 | 0.126  (0.332) |
| gender | 1 Male  2 Female | 48985 | 1.539 |
| Marital status | 1 married  2 separated  3 divorced  4 widowed  5 never married | 47035 | 2.600  (1.790) |
| Age group | 0 <10  1 10-24  2 25-34  3 35-44  4 45-54  5 55-64  6 65+ | 49066 | 3.605  (1.707) |
| Education group | 0 none  1 1-9 years  2 10-19 years  3 20-29 years  4 30 years+ | 49066 | 1.759  (0.554) |
| main occupational activity | 1 paid work  2 education  3 unemployed, looking  4 unemployed, not looking  5 permanently sick or disabled  6 retired  7 community or military service  8 other | 48788 | 3.307  (2.655) |
| Household’s total net income in quintiles | 1 1^st^ quintile (poorest)  2 2^nd^ quintile  3 3^rd^ quintile  4 4^th^ quintile  5 5^th^ quintile (richest) | 37462 | 2.879  (1.4901) |
| feeling about household’s income nowadays | 1 living comfortably on present income  2 coping on present income  3 difficult on present income  4 very difficult on present income | 46471 | 2.066  (0.881) |
| Whether citizen of country | 0 no  1 yes | 49033 | 0.956 |
| Trust in health system back in their original country | Mean of trust by country of birth | 49066 | 5.262  (1.115) |
